# Pre-clinical evaluation of clinically relevant iPS cell derived neuroepithelial stem cells as an off-the-shelf cell therapy for spinal cord injury

**DOI:** 10.3389/fphar.2024.1390058

**Published:** 2024-05-22

**Authors:** Dania Winn, Elias Uhlin, Malin Kele, Ilse Eidhof, Anna Falk

**Affiliations:** ^1^ Department of Neuroscience, Karolinska Institutet, Solna, Sweden; ^2^ Lund University, Department of Experimental Medical Science, Lund, Sweden; ^3^ Department of Medical Biochemistry and Biophysics, Karolinska Institutet, Solna, Sweden

**Keywords:** cell therapy, GMP, spinal cord injury, NES cells, iPS cells

## Abstract

Preclinical transplantations using human neuroepithelial stem (NES) cells in spinal cord injury models have exhibited promising results and demonstrated cell integration and functional improvement in transplanted animals. Previous studies have relied on the generation of research grade cell lines in continuous culture. Using fresh cells presents logistic hurdles for clinical transition regarding time and resources for maintaining high quality standards. In this study, we generated a good manufacturing practice (GMP) compliant human iPS cell line in GMP clean rooms alongside a research grade iPS cell line which was produced using standardized protocols with GMP compliant chemicals. These two iPS cell lines were differentiated into human NES cells, from which six batches of cell therapy doses were produced. The doses were cryopreserved, thawed on demand and grafted in a rat spinal cord injury model. Our findings demonstrate that NES cells can be directly grafted post-thaw with high cell viability, maintaining their cell identity and differentiation capacity. This opens the possibility of manufacturing off-the-shelf cell therapy products. Moreover, our manufacturing process yields stable cell doses with minimal batch-to-batch variability, characterized by consistent expression of identity markers as well as similar viability of cells across the two iPS cell lines. These cryopreserved cell doses exhibit sustained viability, functionality, and quality for at least 2 years. Our results provide proof of concept that cryopreserved NES cells present a viable alternative to transplanting freshly cultured cells in future cell therapies and exemplify a platform from which cell formulation can be optimized and facilitate the transition to clinical trials.

## 1 Introduction

Induced pluripotent stem (iPS) cells can differentiate into all cell types and tissues of the human body, which makes them a promising cell source for regenerative medicine. We have previously developed efficient protocols for the differentiation of iPS cells into neuroepithelial stem (NES) cells (Calvo-Garrido et al., 2021), a multipotent neural stem cell type, capable of differentiating into neurons, astrocytes and oligodendrocytes (Falk et al., 2012). The high proliferative capacity, trilineage differentiation potential, wide plasticity and ability to integrate into the mammalian central nervous system (CNS) (Koch et al., 2009) make NES cells excellent candidates for future cell therapies aiming to repair parts of the CNS.

Spinal cord injury (SCI) is a debilitating condition resulting in lifelong disabilities affecting millions of people globally with very limited treatment options (Csobonyeiova et al., 2019; Shibata et al., 2023; WHO, 2013). The characteristics of a SCI differ depending on the time passed after the initial injury. In the acute phase the inflammation levels are higher compared to the chronic phase, while in the chronic phase the scar formation makes cell regeneration even more difficult (Shibata et al., 2023). Optimal regeneration of SCI will require broad mechanisms of action, depending on the time after injury, including inhibition of glial scar formation, modulation of neurotoxic microenvironment, and providing cell replacement through the differentiation and integration of grafted progenitor cells (Dell’Anno et al., 2018).

Preclinical studies have indicated that the grafting of NES cells into SCI models results in functional recovery (Fujimoto et al., 2012; Xu et al., 2021; 2022) supporting several mechanisms of actions of grafted NES cells, including integration and differentiation combined with promotion of host neuron survival (Dell’Anno et al., 2018; Fujimoto et al., 2012). However, most studies have focused on the sub-acute phase and only a limited number of studies have focused on the chronic phase (Shibata et al., 2023). Grafting of cells to the acute or subacute (3–10 days past injury) phase of injury has been indicated to result in the highest degree of functional recovery (Abbaszadeh et al., 2018). In 20%–30% of cases, patients with a SCI develop post-traumatic syringomyelia which is characterized by an intraspinal formation of a cyst leading to further functional impairment of several body functions. Treatment options are limited, and the development of new strategies is important for reversing and/or decreasing the cyst size in the patients (Xu et al., 2022). In Xu et al., we showed that NES cells decreased the size of the cyst to a larger extent than another type of neural progenitor cells when transplanted in pre-clinical models. However, in those experiments we relied on freshly cultured cells and thus without the possibility of extended quality controls before transplantations (Xu et al., 2021).

Many pre-clinical studies have depended on research-grade cell lines which were derived using integrating vectors for reprogramming, xenogenic and/or undefined media components which have been associated with immunogenicity (Martin et al., 2005), high batch to batch variability (Rodin et al., 2010) and safety concern (tumor formation) (Okita et al., 2007). Such issues pose barriers to clinical transition. Indeed, there are publications reporting studies failing to replicate previous benefits of cell therapies when research-grade cell lines have been replaced with clinical-grade cell lines (Anderson et al., 2017; Marsh et al., 2017). Recently, clinical trials using clinical-grade iPS cell derived neural cells approved for transplantation in SCI (Sugai et al., 2021). To ensure compliance with GMP standards, media without any animal substances or feeder cells must be used (Unger et al., 2008) and the work-flow must follow strict rules to ensure safety and reproducibility (Basnet, 2012). The development of methods to cryopreserve cell therapy doses has been suggested to provide major advantages with regards to the consistency of doses, and the possibility to derive larger cost effective, quality-controlled batches. Cryopreservation would also allow cells to be distributed as an off-the-shelf therapy product, vastly increasing accessibility and enabling grafting of cells in more acute stages of injury (Henchcliffe and Parmar, 2018).

In this study, we provide proof of concept for an off-the-shelf NES cell-based therapy for SCI using cryopreserved doses derived from clinically relevant and GMP cleanroom-produced iPS cell lines.

## 2 Results

### 2.1 Derivation of GMP-compliant human iPS cell line

**FIGURE 1:**
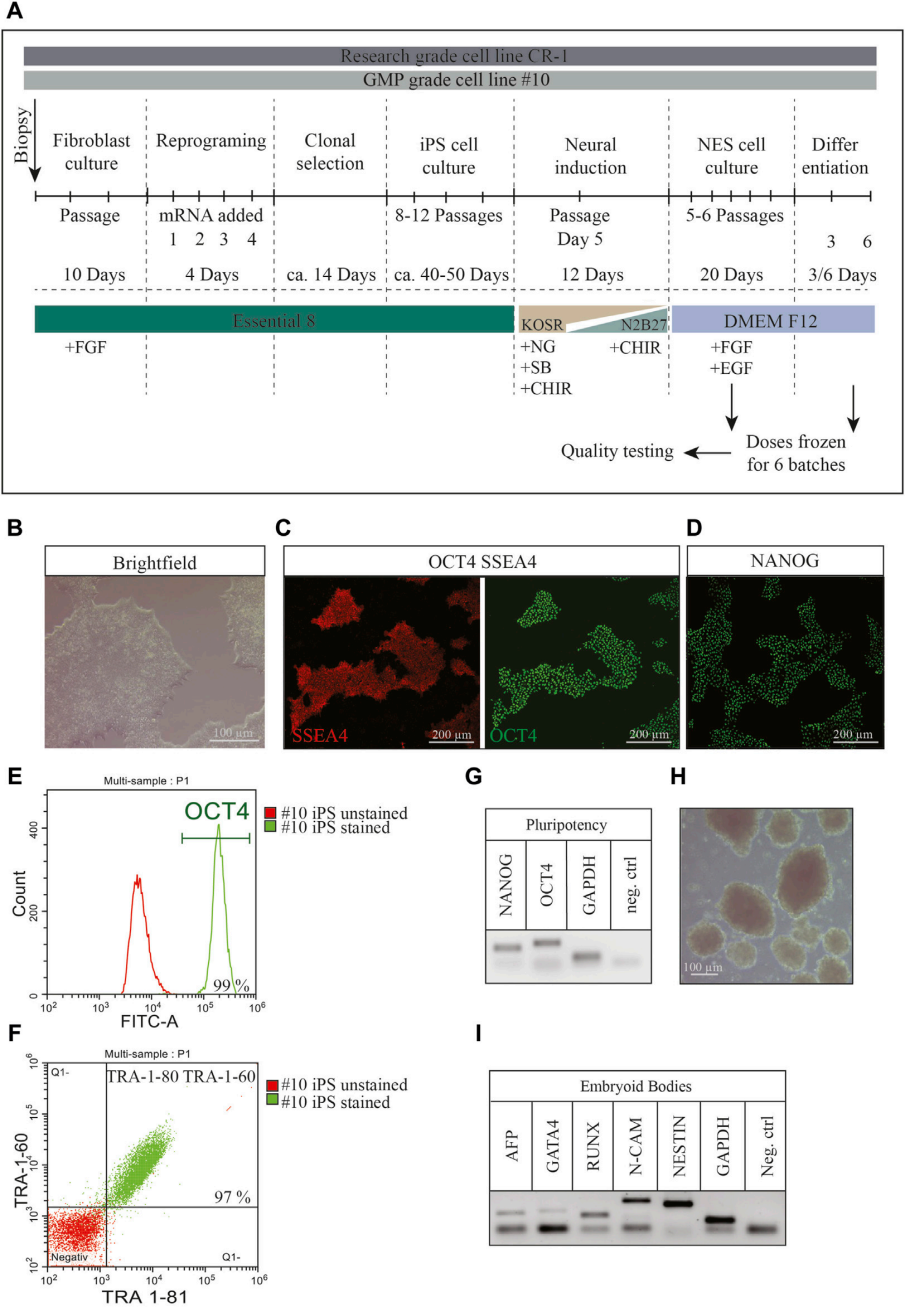
Characterization of newly established GMP-grade iPS cell line #10 **(A)** The experimental overview contains the times and media used for every single step of the protocol to generate frozen cell therapy doses. **(B)** Brightfield pictures of representative monolayer iPS cell colonies. Typical morphological features include compact colonies with homogenous morphology, sharp luminescent edges with cells showing a high nucleus to cytoplasm ratio. **(C)** Immunocytochemistry showing stable expression of pluripotency markers OCT4 (green), SSEA4 (red) as well as **(D)** NANOG (green) in new iPS cell line #10. Scale bar: 200 μm. **(E)** The expression of pluripotency markers was additionally confirmed and quantified by flowcytometry for OCT4 (99% of the cells were positive for OCT4) and **(F)** TRA-1-60 together with TRA-1-80.97% of all cells were double stained for both markers. **(G)** The mRNA expression of pluripotency markers OCT4 and NANOG was further determined by RT-PCR compared to GAPDH and a negative control. **(H)** In order to confirm the differentiation potential of the iPS cell into all three germ layers an embryoid body formation assay was performed and the expression of markers of all three germ layers were shown after 21 days. **(I)** Expression of AFP, GATA4A, RUNX, N-CAM and NESTIN confirmed the existence of cells from all three germ layers in the embryoid bodies.

A human induced pluripotent stem (iPS) cell line, KICRi001-A (hereafter referred to as #10), was derived from a skin biopsy obtained under informed consent from a healthy male donor. This cell line was derived under Good Manufacturing Practice (GMP) compliant rules at the Karolinska Centrum for Cell Therapy GMP facility in approved cleanrooms (Figure 1A). The established iPS cell line met all the quality control criteria for an iPS cell line. Brightfield images showed compact one-layer colonies with sharp luminescent edges with a high nuclei-to-cytoplasm ratio (Figure 1B). All cells maintained a normal 46XY karyotype after reprogramming (Supplementary Figure S1A) and homogenously expressed the pluripotency markers SSEA4, OCT4 (Figure 1C) as well as NANOG (Figure 1D), TRA-1-60 and TRA-1-81 (Figure 1F) as confirmed via immunocytochemistry and flowcytometry (Figures 1E, F). The stable mRNA expression of OCT4 and NANOG was confirmed via RT-PCR (Figure 1G). In order to demonstrate the pluripotent potential of the iPS cells in differentiating into all three germ layers an embryoid body formation assay (Figures 1H, I) was conducted. After 21 days the embryoid bodies demonstrated expression of endoderm, mesoderm and ectodermal markers, confirmed by RT-PCR (Figure 1I). Additionally, the full HLA haplotype and STR profiling were performed (Supplementary Figure S1A, B). Here we show the successful establishment of a clinical-grade GMP compliant iPS cell line derived in clean rooms (A/B-grade) that passed all quality controls for iPS cell lines and differentiated readily into the neural fate (Supplementary Figure S1C, D). This line together with a second iPS cell line (CR-1) were used in this study. CR-1 was generated in a research grade lab, but with the same chemicals and strict protocols as under GMP guidelines (Figure 1A).

### 2.2 Preparation of cryopreserved cell therapy doses

**FIGURE 2:**
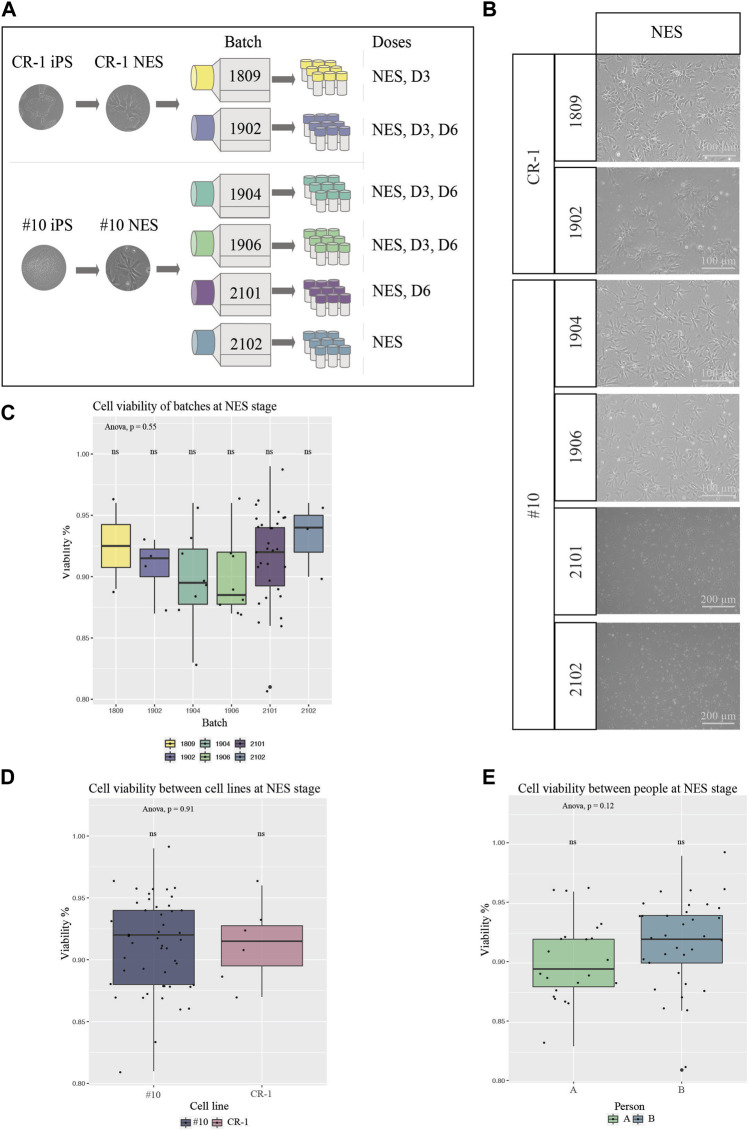
Characterization of cells from all batches for cell therapy **(A)** Schematic overview of experimental procedure. Two clinically relevant cell lines were used to derive six different batches of cell therapy doses. **(B)** BF images of cell therapy doses 24 h post thawing. Cells from all batches and differentiation stages contained high numbers of viable cells with the typical NES cell morphology. Scale bar: 100 μm or 200 μm. **(C)** Viability at thawing was determined by trypan blue viability counting for all six batches at NES stage (1809 N = 2, 1902 N = 4, 1904 N = 8, 1906 N = 8, 2101 N = 30, 2102 N = 3). **(D)** Cell viability compared in-between the two cell lines of origin, NES doses (CR-1 N = 6, #10 N = 49). No statistical significance of cell viabilities was seen in-between batches or cell lines. **(E)** Cell viability compared in-between person A and B for doses at NES stages (A N = 22, B N = 33) showed no significant difference in cell viability between people handling the cells. Significance was tested using a one-way ANOVA and t-test.

One publication has shown the establishment of NES cells under xeno-free conditions via the establishment of embryonic bodies in 3D (Isoda et al., 2016). But the generation of human NES cells from iPS cells using the principles of dual SMAD inhibition (Chambers et al., 2009; Calvo-Garrido et al., 2021) in monolayer using completely defined culture conditions is, to our knowledge, not possible today. Thus, the doses in this study were generated using a standardized monolayer protocol described in the “Material and Methods” paragraph, using close-to-defined conditions. Six batches of cell therapy doses were generated and cryopreserved from the two iPS cell lines described above, both iPS cell lines derived using defined and xeno-free conditions, with CR-1 manufactured in a research lab (research grade) and #10 manufactured in GMP cleanrooms (Figure 2A). Batches 1902, 1904 and 1906 consisted of doses from three differentiation stages; proliferating cells without previous differentiation: NES, cells pre-differentiated for 3 days: D3, and NES cells pre-differentiated for 6 days: D6. Batch 1809 was manufactured to create doses of NES and D3 stages only. Batch 2101 was manufactured in doses of D6 and NES, while batch 2102 was manufactured in doses of NES stage only (Figure 2A). The manufacturing of doses was performed by two different individuals. All doses of cell line CR-1 as well as batches 1904 and 1906 were produced by person A. Doses 2101 and 2102 were produced by person B. It was observed that doses can be frozen for at least 2 years without losing function or quality (data not shown).

### 2.3 Viability of cryopreserved doses

Next, we investigated the quality and reproducibility of our manufactured NES cell therapy doses. One important aspect about the function of cell therapy, aiming at cell integration, is the viability of cells for the transplantation. Cell doses were thawed on tissue culture plates to ensure cells were capable of surviving cryopreservation and imaged with brightfield microscopy 24 h after thawing (Figure 2B). All doses contained high numbers of viable cells capable of adhering to the culture plate, as well as presenting the expected morphology. Cells stained with trypan blue were counted in a hematocytometer to determine the ratio of dead to viable cells (Figures 2C–E and Supplementary Figure S2B–G). We did not detect any significant differences in viability between batches at the NES stage nor between the #10 and CR-1 cell lines (Figures 2C, D). Not surprisingly, a statistically significant difference in viability was observed between the three differentiation stages (NES, D3 and D6) regardless of the cell line (One-way ANOVA, Tukey *post hoc*) (Supplementary Figure S2B) and at D6 (Supplementary Figure S2D). The average viability for each stage of differentiation was 91% for NES, 86% for D3 and 70% for D6. Thus, the further into neuronal differentiation NES cells were the more sensitive the cells were to freezing and thawing. The cell viability differences were not significant between the two NES cell lines (Figure 2D) or the two people handling the cells at NES stage (Figure 2E), showing the robustness of the manufacturing procedure. Taken together, the manufacturing process leads to robust cell batches with reproducible cell viability.

### 2.4 Identity of viable cells

**FIGURE 3:**
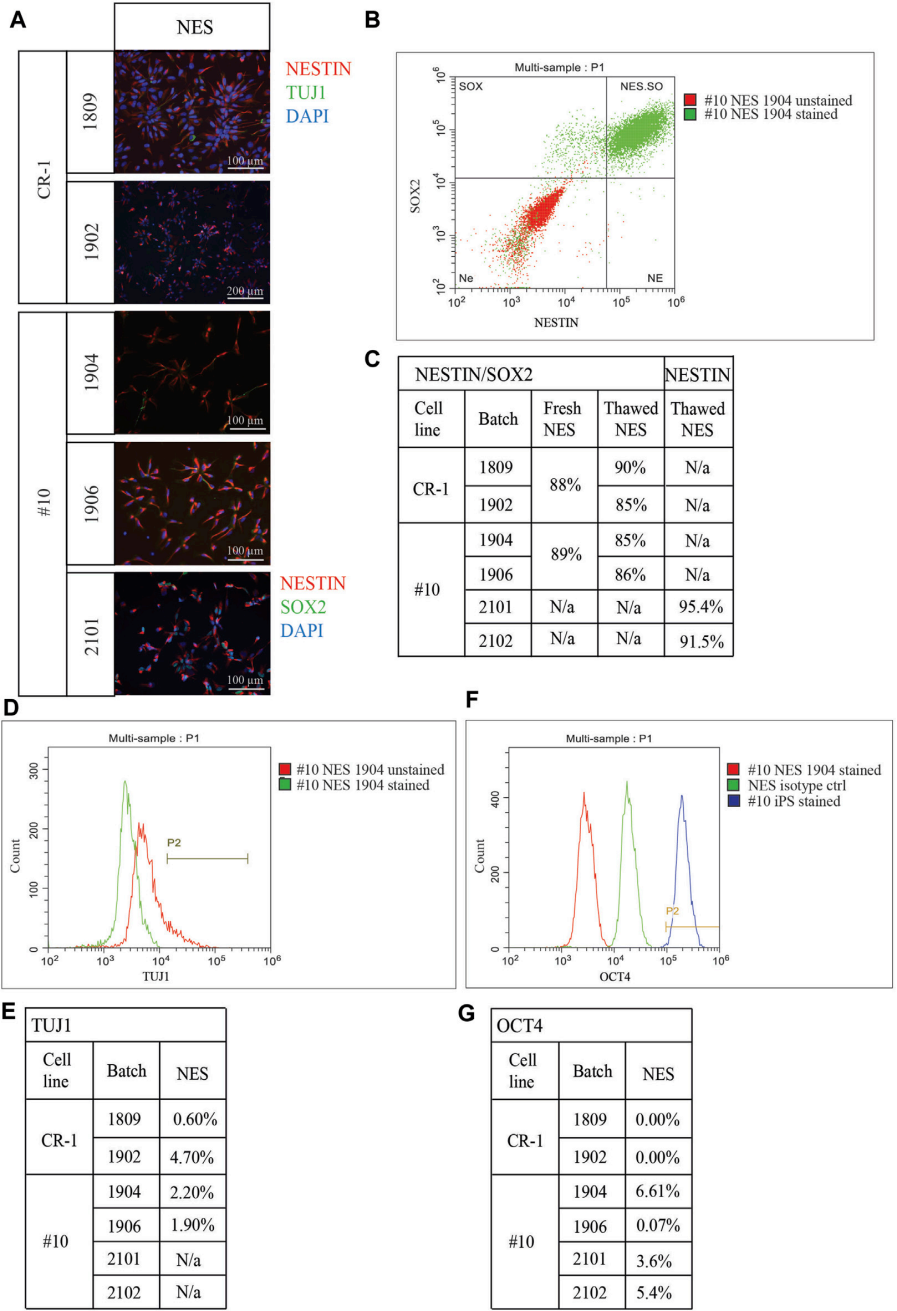
Characterization of cryopreserved doses. **(A)** Immunocytochemistry staining depicting cells 24 h after thawing. Staining for neural stem cell marker NESTIN and neuronal marker TUJ1. NESTIN is expressed in all doses. TUJ1 expression was identified in a small number of cells at NES stage. Doses 2101 and 2102 were stained for NESTIN and SOX2. Both doses contain mainly cells double positive for both NES cell markers. Scale bar: 100 μm or 200 μm. **(B)** Representative flowcytometry plot of cell doses fixed directly post thawing and co-stained for NES cell markers NESTIN and SOX2. **(C)** Table of quantified flowcytometry data for NESTIN/SOX2 co-staining. “Fresh NES” cells from continuous culture were analyzed to ensure frozen cells provide reliable results. Doses showed a decreasing expression profile of NES cell markers the longer differentiation continues. **(D)** Representative flowcytometry plot for neuronal marker TUJ1. **(E)** Table of quantified TUJ1 expression in all batches. Percentage of cells expressing TUJ1 at NES stage ranging from 0.6% in batch 1809 up to 4.7% in batch 1902. **(F)** Representative flowcytometry plot for pluripotency marker OCT4. **(G)** Negligible expression was detected for batches 1809, 1902, 1906, 2101 and 2102. A small percentage of cells in batch 1904 was found to express OCT4 on a small level.

Cell doses were plated on coverslips for immunocytochemistry to assess expression levels of the neural progenitor marker NESTIN and the neuronal marker TUJ1. This was done to determine survival rates and whether thawed cryopreserved cells had retained their cellular identity and functional capacity after cryopreservation (Figure 3A). NESTIN expression was observed in all batches. TUJ1 could be sparsely observed in NES doses but was increasingly observed in D3 and D6 doses (Supplementary Figure S3A, C), indicating that cryopreservation facilitated the survival of both NESTIN positive progenitor cells and neuronal differentiated TUJ1-positive and NESTIN-negative cells. In doses 2101 and 2102, all cells were double stained for NESTIN and SOX2 (Figure 3A).

Flowcytometry was used to quantify the co-expression of the neural stem cell marker NESTIN and the self-renewal marker SOX2. Cryopreserved and thawed NES cells directly from the cryovial were compared to NES cells in culture (fresh NES cells) to determine if reliable marker expression could be acquired from cryopreserved cells without any prior culture (Figures 3B, C). The advantage of such an assay is that the cryopreserved cell doses for transplantation would be assayed directly after thawing without the influence from external factors. Fresh cells from both cell lines (CR-1: 88%, #10: 89%) displayed similar levels of double positive cells to frozen batches (1809: 90%, 1902: 85%, 1904: 85%, 1906: 86%) (Figure 3C). For the doses 2101 and 2102 only the neural progenitor marker NESTIN was analyzed using flowcytometry. In both batches, more than 90% of the cells expressed NESTIN (Figure 3C). Co-staining with NESTIN/SOX2 confirmed the progressive decrease of NES cell markers in the ongoing differentiation in D3 and D6 doses seen in the flowcytometry assay (Supplementary Figure S3B). TUJ1 expression was also quantified using flowcytometry; as expected, the lowest level of expression was seen in NES cells, which increased the further differentiated the cells were (1809: 0.6% and 3.3%, 1902: 4.7% and 6.0%, 1904: 2.2% and 6.9%, 1906: 1.9% and 8.2%) (Figures 3D,E). Mixed results were seen for D6 doses, in two batches a slightly lower expression compared to D3 was observed (1904: 6.8% and 1906: 6.4%) while in one dose the expression of TUJ1 was higher in the later differentiation stage D6 (1902: 12.5%) (Supplementary Figure S3C).

Expression of a pluripotency marker OCT4, which should be downregulated when iPS cells differentiate, was analyzed in all cell therapy doses (Figures 3F, G) and compared to an isotype control and the #10 iPS cell line. Flowcytometry of cell doses revealed low levels of OCT4 expression. However, the levels observed were much lower than in the compared iPS cell line. Negligible expression of OCT4 was observed in batches 1809, 1902, 1906 (between 0.0% and 0.07%) while a slightly higher expression was observed in doses from batches 1904, 2101 and 2102 (between 2.7% at D3 and 6.61% at NES stage) (Figure 3G; Supplementary Figure S3D).

In summary, the cells from all batches expressed the typical NES cell markers at expected levels while showing low levels of OCT4 expression. Batches of NES cells can be manufactured, frozen and thawed without losing functionality.

### 2.5 Functional assessment of cell therapy doses

**FIGURE 4:**
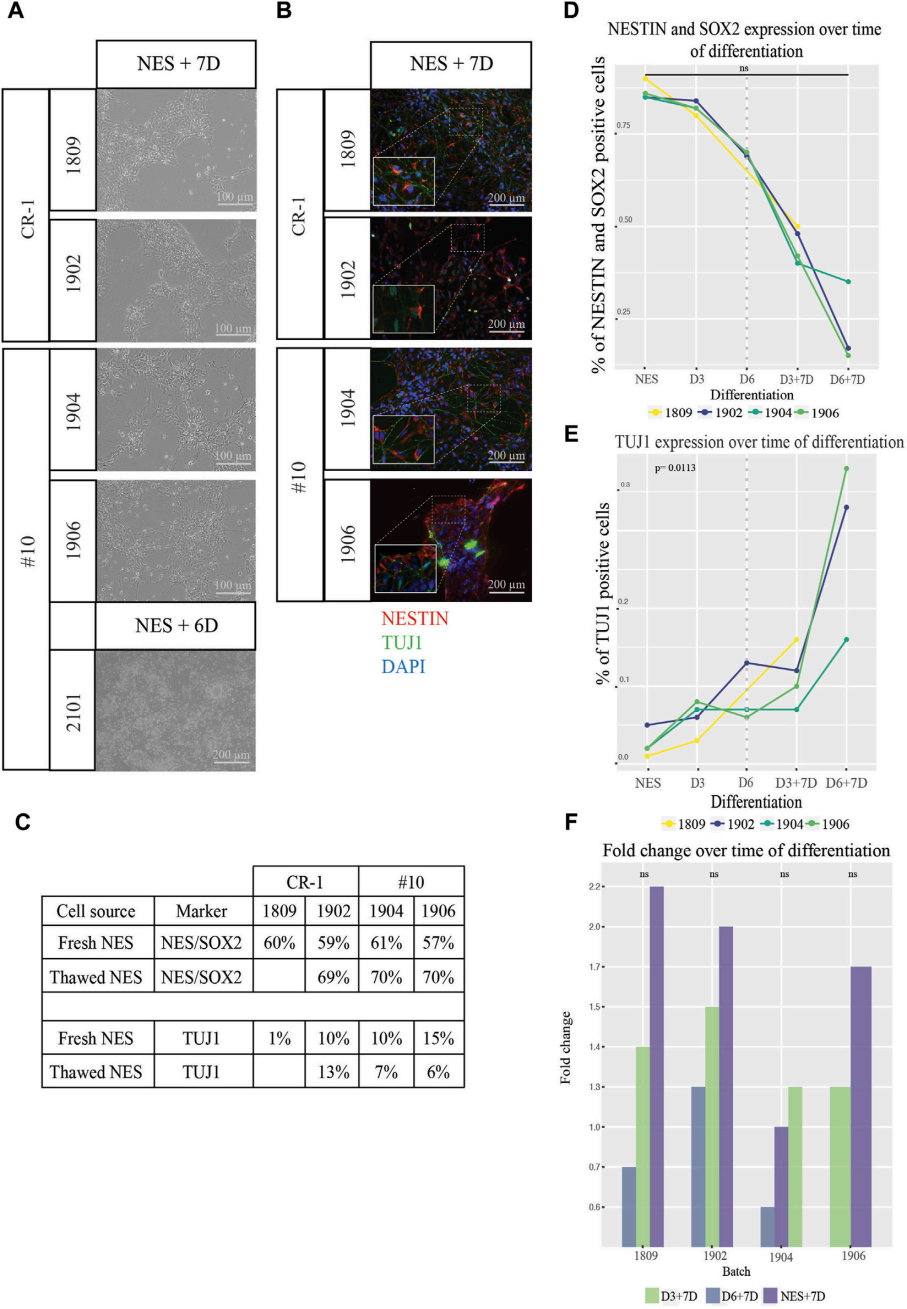
Functional testing of differentiation and proliferation potential of cell therapy doses. **(A)** Brightfield images of NES cell doses after 6–7 days in differentiation media. Cells remained viable over the whole time period and displayed increasing neuronal morphology and a higher degree of axonal projections the higher the stage of differentiation. **(B)** Expression of the markers NESTIN (red) and TUJ1 (green) confirmed by immunocytochemistry. Cropped pictures show zoom-in of relevant region indicated by dotted lines for each picture. Scale bar: 100 μm or 200 μm. **(C)** Percentage of cells expressing NESTIN and SOX2 as well as TUJ1 when cells from fresh cell culture were compared to thawed batches. Values were comparable between the two conditions fresh vs. frozen cells for all markers. **(D)** Flowcytometry analysis of NESTIN/SOX2 revealed a continuous loss of NESTIN/SOX2 double positive cells and an increase of **(E)** TUJ1 positive cells as differentiation progressed. Values left of the dotted line indicate cell doses analyzed directly after thawing and values right of the dotted line indicate progression for doses thawed and differentiated for 7 additional days *in vitro*. Cells differentiated from NES cells without prior cryopreservation compared to thawed D6 cells appear to exhibit slightly higher levels of differentiation marker TUJ1 expression and a lower proportion of cells double positive for the NES cell markers NESTIN and SOX2. To test if there was in increase in the marker levels the significance was tested between the growth slopes of the curves between the different batches of cells. The slopes were not significantly different between the different batches over the time of differentiation for NESTIN/SOX2 double staining (*p* > 0.05) while for TUJ1 **(E)** an exponential growth curve fitted for three out of four batches. These three batches did not differ significantly (*p* > 0.05) while the fourth one differed significantly from the other three (*p* = 0.0113). **(F)** Fold change in cell numbers after doses were cultured in differentiation media for 7 days. An increase in cell numbers was observed for both NES and D3 doses and a decrease in numbers for D6 doses. A regression lines was added between the data points of NES + D7, D3+D7 and D6+D7 for each batch. The slopes between the different regression lines for each batch of cells didn´t differ significantly from each other. A linear regression model was the best fit for this analysis (*p* > 0.05).

To ensure that the cell doses retained the capacity to differentiate, we investigated the *in vitro* differentiation capacity. Doses from each batch and differentiation stage were plated on tissue culture plates in differentiation medium and allowed to differentiate for 6–7 days to investigate the differentiation potential after freezing and thawing. Cells from all batches survived and differentiated with the expected change in cell organization and morphology *in vitro* (Figure 4A). Immunocytochemistry staining for NESTIN, and TUJ1 showed higher levels of TUJ1 expression, axonal projections, and cellular organization the further differentiated the doses (D3 and D6) were before freezing (Figure 4B; Supplementary Figure S4B). Hence, the ability of cell doses to differentiate does not seem to have been limited by cryopreservation.

Quantifications of the expression of cell type specific markers by *in vitro* differentiated cell doses was tested using flowcytometry (Figures 4C–F). The percentage of cells expressing both NES cell markers NESTIN and SOX2, declined gradually (right of dotted line) according to the trend observed before freezing (left of the dotted line) (Figure 4D). NESTIN/SOX2 expression in cells with the longest differentiation (D6+7D) showed as little as 15%–35% of double positive cells. 33.3% of cells expressed the neuronal marker TUJ1 for batch 1902, 16.0% for batch 1906 and 37.5% for batch 1904 at D6+7D (Figure 4C).

The capacity of both progenitor cells and more differentiated TUJ1 positive neurons to survive cryopreservation and the continued differentiation post thawing is encouraging.

We further asked the question whether survival of cells at the different differentiation stages is equal depending on the expression of the different markers. The lower viability for D6 doses suggests otherwise. Cells from cryopreserved D6 doses were analyzed using the flow-cytometer and compared to NES cells kept in a continuous culture, differentiated for 6 days or without prior cryopreservation (Figure 4E). Cells from fresh differentiations showed lower expression of NESTIN/SOX2 compared to cryopreserved and thawed doses. Similarly, the batches 1904 and 1906 showed lower TUJ1 expression than corresponding fresh batches. The exemption being batch 1902 where a slightly higher expression of NESTIN and SOX2 was discovered. Taken together this indicates that differentiated cells survive cryopreservation but are more sensitive than undifferentiated NES cells.

To establish a rough understanding of how much cell growth would be expected post grafting, cell doses were thawed *in vitro* and allowed to grow in differentiation culture for 7 days (Supplementary Figure S4A). The cell numbers were determined using a hematocytometer and fold changes of cell numbers were calculated. The NES doses showed the highest fold change increase, on average across batches 1.7 times compared to 1.3 for D3 and 0.7 for D6 (Figure 4F). All in all, this shows that frozen cell batches maintained their differentiation and proliferation potential upon thawing *in vitro.*

All together the two different cell lines, CR-1 (research grade) and #10 (GMP grade) did not differ significantly from each other nor from previously derived and published NES cell lines. Showing that the GMP grade lines remains with the same functional properties when compared to the research grade lines.

## 3 Discussion

In this study, we demonstrate a process to manufacture, cryopreserve and characterize iPS cell derived NES cell therapy doses with consistent good quality for preclinical trials and potential for treating humans.

The main purpose of preclinical trials is to elucidate efficacy and safety of cell doses. Despite the apparent success of previous NES cell transplantation studies, only recently iPS cell derived neural stem cells have been approved for clinical trials in SCI. For this, NES cells get manufactured and transplanted as 3D cell aggregates free floating in culture (neurospheres) (Isoda et al., 2016; Sugai et al., 2021) making it harder to completely standardize the cell manufacturing and transplantation with a risk for inconsistent results. Difficulty in producing consistent data supporting safety and efficacy especially when using fresh cell therapy doses is a potential hurdle for clinical translation of cell therapy. The use of fresh GMP grade cells is one factor limiting the use of cell therapy due to the limited time available for characterizing the cells prior to grafting (Henchcliffe and Parmar, 2018). In addition, a consistent supply of fresh cells for transplantations relies on the existence pf advanced GMP clean rooms and highly qualified staff in facilities close to the patients. Furthermore, retaining cells in culture for a long time increases the cost and the use of resources, which decreases sustainability. On top of that, research has demonstrated that cells subjected to multiple passages are more prone to develop karyotypic abnormalities (Sugai et al., 2021). Thus, the use of frozen off-the-shelf cell therapy products would be the safest, most efficient, and sustainable way to take iPS cell derived cell therapy into the future.

Further facilitating clinical translation of our study, cell doses were prepared from two standardized iPS cell lines, which have been derived completely xeno-free and in chemically defined conditions. They have been reprogrammed using integration free mRNA technology. In addition, cell line #10 has been manufactured in accordance with GMP in approved cleanrooms (A/B-grade) in a manor suitable for clinical trials (Unger et al., 2008) while CR-1 was generated as a research grade cell line, but using the same rigorous protocol and GMP compliant chemicals for its generation. These cell lines were derived with minimal adaptations to the manufacturing process to ensure efficacy, as previous studies have shown that significant adaptations can lead to a loss of efficacy during the transition to clinical trials (Anderson et al., 2017; Marsh et al., 2017). By the extensive characterization of the GMP-grade iPS cell line #10 we showed that the generation of an iPS cell line under the strict GMP rules in clean rooms results in a high-quality cell line with similar potency in creating frozen cell therapy doses as a research grade line. Proving the robustness of our protocols and competence of our cell experts.

Even though not significant there is a tendency for a difference in cell viability between the persons handling the cells. This shows that even with a robust and standardized protocol the viability of cells after thawing is slightly different depending on the person manufacturing the cell therapy doses. Despite, not significantly different in our study this variation underlines the need for automized processes for manufacturing, thawing and preparing doses for cell therapy to mitigate even the slightest differences and errors introduced by humans handling the cells.

The cryopreservation of doses facilitates extensive quality control testing before cell grafting and opens up new opportunities to standardize and optimize cell doses in preparation for human clinical trials. Previous preclinical studies of transplantation of NES cells in SCI could not apply the same rigorous characterization of cell therapy doses before grafting and were limited to a more general characterization of cell lines rather than the doses due to the use of fresh cells (Dell’Anno et al., 2018; Fujimoto et al., 2012). This limits the potential to pinpoint the optimal characteristics and quality controls of cells pre-transplantation to predict the best functional recovery. Quality control marker testing in batches of cell therapy doses, predicting the best functional outcome of the cell therapy, needs to be developed and could in the future also act as release criteria approved by the medical agencies. Our method using a monolayer, standardized and defined protocol for deriving frozen cell therapy doses will allow us to thoroughly characterize the cells with single cell RNAseq and single cell proteomics to identify gene and protein panels predicting efficacy and potency in pre-clinical transplantations.

Our frozen cell therapy doses show constant safety after transplantation into animals and no overgrowth or teratoma formation was observed, despite the very low expression of OCT4 in the pre-transplantation QC (Xu et al., 2022). Other studies using iPS cell based cell therapies use a more stringent pluripotency marker like NANOG, to determine the percentage of pluripotent cells in the cell therapy doses (Emgård et al., 2014; Kirkeby et al., 2023) or grow the cell doses in iPS cell conditions to observe the formation of colonies, which would suggest residual iPS cell in the cell therapy doses (Sugai et al., 2021). OCT4 is expressed in pluripotent stem cells but has also been shown to be expressed in neural stem cells (Chin et al., 2009). For future experiments and QCs a different set of marker genes might be needed to reliably show that no pluripotent cells remained in the cell therapy doses pre-transplantation.

We showed the differentiation potential of NES cells towards the neuronal lineage by the expression of the neuronal marker TUJ1 after 7 days of *in vitro* differentiation for all batches. Interestingly the outlying batch 1904 which had the lowest TUJ1 expression also retained the highest proportion of NESTIN/SOX2 positive cells during differentiation and the highest OCT4 expression at all differentiation stages. Obtaining this type of information would only be possible when using frozen cell therapy doses with time for extensive QC compared to transplantations using freshly produced cells and thus less time for QCs.

Data from literature has shown that NESTIN expression remains in grafted neural precursors cells over 20 weeks *in vivo* (Emgård et al., 2014). Even though this data is not from NES cell transplantations, we were still curious to see if pre-differentiation of NES cells for 3 days or 6 days would enhance the differentiation of cells post-thawing. Not surprisingly, viability of differentiated cells post-thawing was proven to be lower for D3 and especially D6 doses compared to NES cells, but it was possible to deliver doses at targeted cell numbers. From previous experiments we know that the cells become more and more sensitive to handling the longer they differentiate towards the neuronal fate. The *in vitro* differentiation showed that D6 doses subjected to additional 7 days of *in vitro* differentiation after thawing only retain around 20% NESTIN/SOX2 compared to more than 70% in undifferentiated NES cells. Proving that NES cells differentiate relatively fast towards the neuronal fate when cultured in differentiation conditions. It is important to remember that the environment *in vivo* may be drastically different than the conditions *in vitro* tested here. The *in vitro* experiments merely serve as proof of the functional capacity of the cells to differentiate despite having been subjected to cryopreservation at different stages. Furthermore, we did not detect any beneficial effect neither on the cell differentiation outcome nor functional effects of pre-differentiated doses compared to NES doses. This led to the decision to focus on producing and transplanting frozen NES cell doses after the first rounds of transplantations with the earlier batches of cell therapy doses (data not shown).

One possible effect of transplanted cells into the spinal cord is that these cells can restore the regeneration potential of injured neural cells by differentiating into various cell types important for the proper function of the nervous system (Kawai et al., 2021). Analysis of cells from this study after *in vivo* differentiation could indeed show several beneficial effects. Cells differentiated towards neurons, astroglia and oligodendrocytes in addition to staying in the NES state. Importantly, there was no sign of tumor or teratoma formation or cells overgrowing in the rats. Even further, cell transplantation had positive effects both on the formation of a cyst in a sub-acute phase (1 week after SCI) as well as the cyst size in the chronic phase of a SCI (10 weeks after SCI). (Xu et al., 2022). Overall, in Xu et al., we could show that the transplanted cells survived the transplantation, were able to proliferate and differentiate *in vivo* and had beneficial functional effects after spinal cord injury.

The shelf life of doses is important to track. One of the major benefits of cryopreserved doses described here is that they can be offered as an off-the-shelf therapy and even stored by local hospital pharmacies. NES cells can routinely be cryopreserved for several years with conserved viability (data not shown). Although mere survival of the culture may be sufficient for research, more stringent demands will be put on cell therapy doses. The oldest doses thawed for QC in this study had been cryopreserved over 3 years prior to QC testing. These cells passed QC without any observed deviations with regards to either viability, differentiation capability or marker expression. This indicates that a shelf-life of doses of around 1 year would not be inconceivable.

The success of future therapies will depend not only on the ability of regenerative therapies to provide functional recovery but also requires the benefit of therapies to offset costs, generally estimated to be very high for cell therapies (Simaria et al., 2014). The ability to cryopreserve cells means large cost-effective batches can be manufactured. Cell lines and doses can take months to prepare, relying on fresh cells may eliminate the possibility of HLA-matching cells to the recipient, as well as performing safe and extended QCs, which would complicate immediate treatment in the acute or subacute phase of injury and require highly skilled cell culture specialists and GMP level cleanrooms in close vicinity of the clinics. The described hurdles will drastically limit the availability of therapies that might not reach all patients in need. It has been shown that the correct timepoint of transplantation when it comes to SCI is vital, since the environment around the lesion is changing drastically as time passes (Shibata et al., 2023). Availability of quality-controlled cell doses frozen and ready to use at any given time point increases the chances of transplantation at the best possible time window compared to cell therapies freshly generated. Additionally, generating and freezing ready-to-use cell doses makes it possible to manufacture big batches with the same high-quality doses under standardized culturing protocols limiting batch effects that can appear when cell doses are generated fresh for every treatment.

In conclusion, we show that NES cells and pre-differentiated NES cells can survive cryopreservation without losing their functional properties and characteristics. Generated batches manufactured using our process show consistent viability and identity despite doses having been derived at different occasions and manufactured using different cell lines and by different people. We also show that these cell doses remain typical in their functional ability to differentiate *in vitro,* and some effects could be shown *in vivo* (Xu et al., 2022). We believe in the importance of our study for laying the foundation of manufacturing off-the-shelf iPS cell derived efficacious NES cells therapy products in a sustainable, safe and standardized manner to reach beyond clinical trial and the possibility to treat all patients in need.

## 4 Materials and Methods

### 4.1 Establishment of primary fibroblast culture from dermal biopsy

2 mm dermal biopsies were collected from healthy donors, mechanically dissected and enzymatically digested using 0.1% recombinant dispase II solution, Life Technologies #17105-041, prior to incubation at +4°C for 16 h. 0.1% recombinant collagenase I solution (Thermo Fisher Scientific, #17100017) was prepared in PBS (Thermo Fisher Scientific, #14190169), added to the biopsy digest and incubated at +37°C for 16 h. The remaining digest was plated on 12 well tissue culture plates coated with CTG521 (BioLamina, #CT521) in Essential 8 medium (Thermo Fisher Scientific #A15169), +1:100 Penicillin-Streptomycin, (Thermo Fisher Scientific, #15140-122), +20 ng/mL bFGF, (Thermo Fisher Scientific, #13256029). The cells were passaged 1:3 once they reached confluency as follows: cells were washed with PBS and 1 mL of TrypLE Select (Invitrogen, # 12563) was added for 5 min at 37°C. After that 5 mL of E8 media (Thermo Fisher Scientific, #A15169) were added to the flask and the media with pipetted up and down to dissociate the cells from the culture flask. Cells were transferred to a falcon tube and centrifuged for 3 min at 300 g. The supernatant got discarded and the cells resuspended in 12 mL of media. 4 mL of cell suspension got added to a fresh T25 flask coated with CTG521. The media was changed every other day. The fibroblasts were cultured for two to three passages before they were reprogrammed.

### 4.2 Reprograming of dermal fibroblasts

Dermal fibroblasts were seeded on CTG521 coated 24 tissue culture plates and transfected by mRNAs for Oct4, Sox2, Klf4, cMyc, Nanog, Lin28, using the StemRNA™-NM Reprogramming Kit, (Stemgent/Reprocell, #00-0076) for four consecutive days. Colonies were clonally expanded in Essential 8 medium on CTG521 (Biolamina, #CT521) coated tissue culture plates. Emerging colonies were manually picked after 12–16 days and clonally expanded in Essential 8 Medium on CTG521 coated tissue culture plates. Cell clones were enzymatically passaged as single cells for consecutive passages using TrypLE Select (Invitrogen, #12563) and plated on CTG521 laminin coated tissue culture plates in Essential 8 medium supplemented by ROCK Inhibitor, (Millipore, #SCM075) once the clones reached a size of 5 mm. After passaging they reached around 80% confluency.

### 4.3 iPS cell maintenance

For detailed description of iPS cell culture see Calvo-Garrido et al., 2021. In short: iPS cells were cultured on Lam521 coated tissue culture plates in Essential 8 medium until they reached a confluency of 80%–90% before they were passaged as single cells using TrypLE Select (Invitrogen, #12563). 10 μM ROCK Inhibitor (Millipore, #SCM075) was added to the culture medium at day of passage. Cells were seeded at 17,500–25,000 cells/cm2 and the media was exchanged every day until the cells were ready to be split again.

### 4.4 Embryonic body formation assay

Cells were single cell passaged and transferred to suspension culture 6 well plates in DMEM/F12 (Life Technologies, #31331-028) with 5% KnockOut Serum (Life Technologies, #10828-028), 1:100 Non-essential Amino Acids x100 (Life Technologies, #11140-076), 0,2% 2-mercapthoethanol 50 mM (Life Technologies, # 31350-010), 1:100 Penicillin-Streptomycin (Gibco, #15140-122), and 10 μM ROCK inhibitor (Millipore, #SCM075). After 7 days aggregates were transferred to a 0.1% gelatin coated 12 well tissue cultured plate and cultured for 14 days. Embryonic bodies were fed every 3 days with the media mentioned above.

### 4.5 STR profiling

Cell authentication of fibroblasts and iPS cells were performed by Eurofins Genomics forensic, Ebersberg, Germany, using PowerPlex 21 kit, (Promega, #DC8902).

### 4.6 HLA-typing

HLA typing by Next-generation Sequencing was performed by Clinical Immunology, Karolinska University Hospital, Solna, Sweden.

### 4.7 Karyotyping

The G-banding analysis was performed at Ambar, Barcelona, Spain.

### 4.8 RT-PCR

Total RNA was extracted using RNeasy plus mini kit, (Qiagen, #74134) and cDNA synthesized using iScript™ Advanced cDNA Synthesis Kit, (Biorad, #1725038). 0.5 μg of template was loaded with Phusion high-fidelity DNA polymerase, (Thermo Fisher Scientific, #F530S), PCR and run for 32 cycles. Both positive control (GAPDH) and Negative control with (GAPDH primer but without cDNA template).

### 4.9 Neural induction of iPS cells and capture of NES cells

The neural induction protocol (Calvo-Garrido et al., 2021) was adapted from Chambers et al., 2009; Chambers et al., 2009). Briefly: iPS cells were plated on Lam521 (Biolamina, #LN521) coated tissue culture plates (dilute Lam521 1:20 in PBS) in Essential 8 medium and incubated at 37°C for 24 h. Medium was changed to knockout-serum-replacement (KOSR) medium (79% DMEM/F12+GlutaMax (Gibco, #31331-028), 1:5 KnockOut Serum Replacement (Gibco, #10828028), 1:100 Non-essential Amino Acids (Gibco, #11140-076), 1:550 2-mercapthoethanol (Gibco, #31350-010), 1:100 Penicillin-streptomycin (Gibco, #15140-122) supplemented fresh everyday by 10 µM of SB431542 (StemCell Technologies, #72232), 500 ng/mL of Noggin (PeproTech, #120-10C) and 3.3 µM CHIR (StemCell Technologies, #72052)). KOSR medium was changed every 24 h for the first 4 days of induction. Cells were passaged 5 days after plating and 125,000 cells were seeded in a new well of a Lam521 coated 12-well tissue culture plate. The media after splitting got supplemented with 1:1000 ROCK inhibitor (Millipore, #SCM075). Starting after passage at day 5, only CHIR (3.3 µM) and Noggin (500 ng/mL) were added to the medium (SB421542 was not added) and the KOSR medium was mixed with an increasing level of N2B27 medium (48% DMEM/F12 GlutaMax (Gibco, #31331-028), 48% Neurobasal (Gibco, #21103-049), 1:500 2-mercapthoethanol, 1:200 N2 supplement (Gibco, #17504-044), 1:100 B27 supplement (Gibco, #17502-048) and 1:100 penicillin-streptomycin (Gibco, #15140-122)). On day 5 and 6 the media contained 75% KOSR media and 25% N2B27 media. Day 7 and 8 were 50% KOSR and 50% N2B27 media and day 9 and 10 the media was made up by 25% KOSR and 75% N2B27 media. From day 11 the only media used was N2B27 media supplemented with 1:3000 CHIR (no NG or SB added). On day 12, the cells were passaged with 1*10^6^ cells per well in a 12-well tissue culture plate coated with poly-L-ornithine (100 μg/mL) (Merck, P3665, #27378-49-0) and Laminin 2020 (2 μg/mL) (Sigma-Aldrich, #114956-81-9) and cultured as NES cells. For the first passage 10 µM ROCK Inhibitor (Millipore, #SCM075) is added fresh to the NES medium.

### 4.10 Culture of NES cells

NES cells were cultured on tissue culture plates coated with 100 μg/mL poly-L-ornithine (Merck, P3665, #27378-49-0) for 2 h, washed with PBS followed by coating with 2 μg/mL laminin 2020 (Sigma-Aldrich, #114956-81-9) overnight. Cells were fed daily with NES medium (DMEM/F-12 Glutamax (Gibco, #31331-028), 1:100 penicillin-streptomycin (Gibco, #15140-122), 1:100 N2 supplement (Thermo Fisher Scientific, #17502001), 1:1000 B27 supplement (Thermo Fisher Scientific, #17504044), 10 ng/μL bFGF (Thermo Fisher Scientific, #13256-029), and 10 ng/μL EGF (PeproTech, #AF-100-15)). Cell culture media was changed daily. Cells were passaged as single cells using TrypLE Select 1x once they reached 90% confluency.

### 4.11 Manufacture of cell therapy dose batches

0.5 *10^6^ NES cells from either NES cell line CR-1 or #10 were seeded in 100 μg/mL poly-L-ornithine and 2 μg/mL laminin2020 coated T75 flasks (VWR, #VWRI734-2313_P) (D-1) and cultured in NES medium for 24 h. The cells in 2 of the flasks intended to be cryopreserved as NES cell therapy doses were kept in NES medium with daily media changes for an additional 48 h before harvest. The cells in the remaining 6 flasks were changed to diff media (DMEM/F-12 Glutamax, 1:100 penicillin-streptomycin, 1:100 N2, 1:100 B27) and allowed to spontaneously differentiate with media changes every 48 h. The time point of media change to diff media was counted as D0. 72 h after the first media change D3 doses were harvested. D6 doses were harvested 144 h after the medium was changed to differentiation medium.

### 4.12 Harvest and cryopreservation procedure of cell therapy doses

At the day of harvest cells were passaged as single cells using TryPLE select and incubated in 37°C for 3 min. Wash medium was added (DMEM-F12 + 0.2% BSA) and cells in suspension counted and centrifuged at 300 g. The cell pellet was resuspended in +4°C cold PSC cryopreservation medium (Thermo Fisher Scientific, #A2644601) with a concentration of around 2*10^6^ cells/mL. Approximately 1*10^6^ cells were added to each cryovial. On average one batch yielded around 17 doses per differentiation stage. Pellets of 2 × 4*10^6^ cells were collected from each stage and stored in −80°C. After adding cell therapy doses to the cryomedia they were immediately transferred to a CoolCellLX container (Corning) and stored at −80°C for 24 h and later transferred to liquid N2 for long term storage.

### 4.13 Thawing and preparation of cell therapy doses pre-injection

Cell doses were transferred from liquid N2 to dry ice for transportation, thawed in a water bath at 37°C for 30 s and washed using wash medium at room temperature. Cells were centrifuged and the cell pellet was resuspended using fresh wash medium. To determine the ratio of viable cells in the suspension, dead cells were stained using trypan blue (Thermo Fisher Scientific, #15150061) and the cells were counted using a hematocytometer. Cells were centrifuged and the pellet resuspended in injection medium (DMEM/F12 + 1% penicillin-streptomycin) corresponding to a concentration of 100,000 viable cells/mL.

### 4.14 Viability assessment of doses

The cell therapy doses were thawed as indicated above and stained using trypan blue. The number of stained cells and the total number of cells were obtained using a hematocytometer.

### 4.15 Statistical assessment

The effect of various parameters on cell viability was calculated using One-way Anova and Tukey’s *post hoc* test or a t-test to determine statistical significance of observations. For Figure 4D, F a linear regression model was chosen. The slopes for every batch were compared. For E) it was tested if the same exponential curve would fit for all batches. This analysis showed a significant difference for one of the tested batches (1904) to the other three batches (Extra sum-of-squares F test). For figure F the significance of the difference in the slope of a regression line through the data points at each time point for each batch was tested.

### 4.16 Flow cytometry

Cell therapy doses were thawed and centrifuged in stain buffer (BD Pharmingen #5546) and immediately fixed and permeabilized using the Foxp3/transcription Factor fixation/permeabilization kit (Thermo Fisher Scientific, #00-5521-00) according to manufacturer’s instructions. Cells were incubated in permeabilization buffer with conjugated antibodies diluted 1:500 (NESTIN PER-CyP-CY5.5 (BD Bioscience, #561231) SOX2 A488 (BD Bioscience, #560301), OCT3/4 A488 (BD Bioscience, #561628), or TUJ1 (A488 BD Biosciences, #560338)) and incubated at +4°C for 30 min. Following that, cells were washed and resuspended in stain buffer. The FACS analyses were acquired on the CytoFLEX flow cytometer from Beckman.

### 4.17 Immunocytochemistry

Cell therapy doses were thawed and counted as described above and 80,000 cells were plated in poly-L-ornithine and laminin2020 coated cell culture plates containing one coverslip each in either NES or differentiation media (for D3 and D6 doses). The cells were incubated for 24 h in 37°C 5% CO2 and fixed in 4% formaldehyde. Cells were then permeabilized at room temperature for 60 min using permeabilization buffer (PBS +10% FBS +0.2% TritonX100 (Merck, X100)). Primary antibodies (mNestin (Sigma, #MAB5326), rbTUJ1 (Nordic Biosite, #802001)) were diluted 1:200 in permeabilization buffer and incubated in +4 °C overnight. After 4 washing steps the secondary antibodies (donkey anti-rabbit A488 (Invitrogen, #A21206) or goat anti-mouse (Life Technologies, #A116004)) were diluted 1:500 in permeabilization buffer and the cells were incubated for 60 min at RT. After washing with PBS three times the nuclei of cells were stained using DAPI (Sigma-Aldrich, #D9542) diluted 1:5000 in PBS and for 10 min at RT. Coverslips were washed in PBS and mounted onto microscope slides using fluorescence mounting media (Agilent Dako, #S3023).

## Data Availability

The original contributions presented in the study are included in the article/Supplementary Material, further inquiries can be directed to the corresponding author.
