# Supplementary figures and images for: Pre-clinical evaluation of clinically relevant iPS cell derived neuroepithelial stem cells as an off-the-shelf cell therapy for spinal cord injury
Source: Front Pharmacol. 2024 May 22;15:1390058. doi: 10.3389/fphar.2024.1390058 (PMC11150580; doi:10.3389/fphar.2024.1390058)

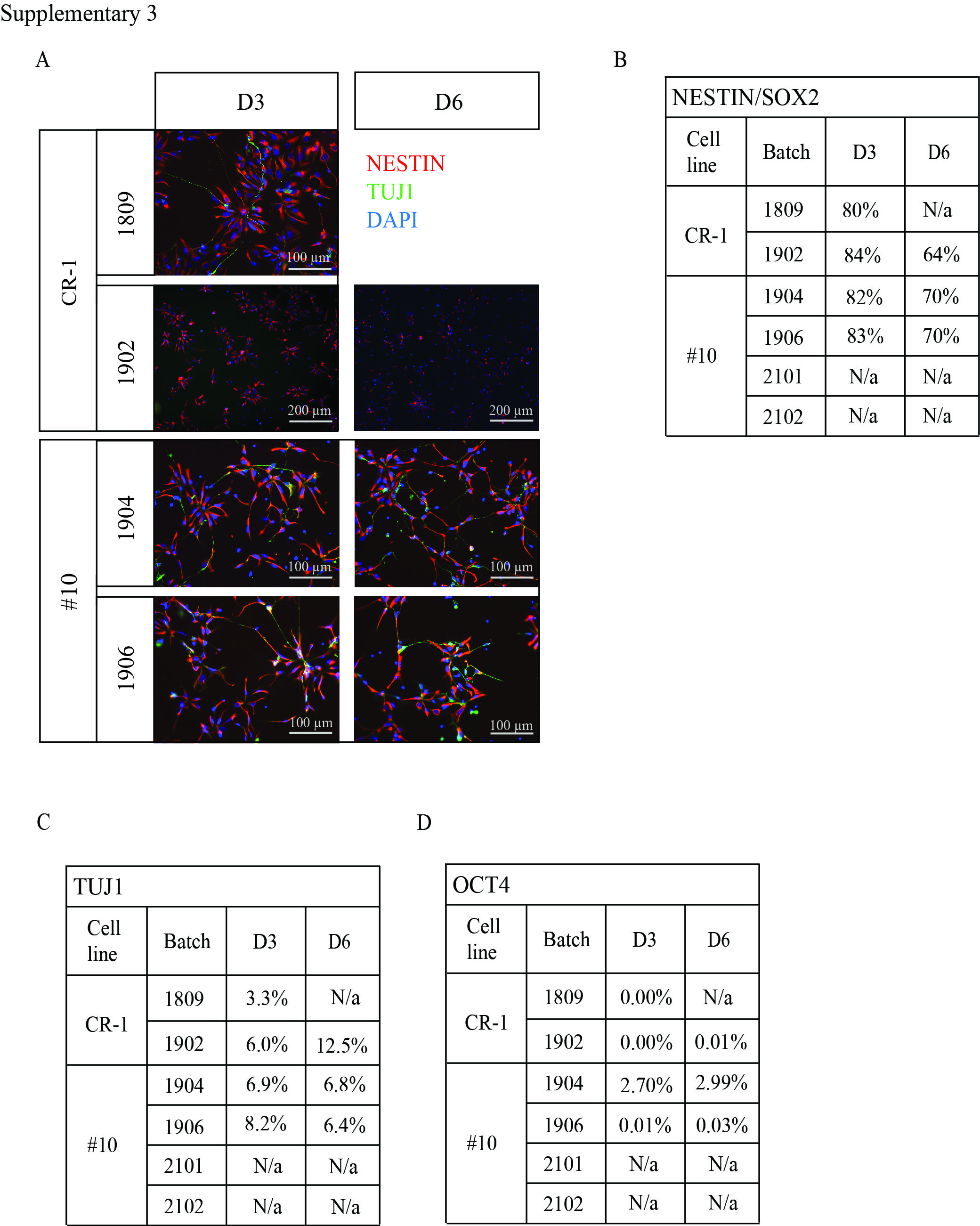

Supplement: Supplementary file 1 [file Image3.JPEG]

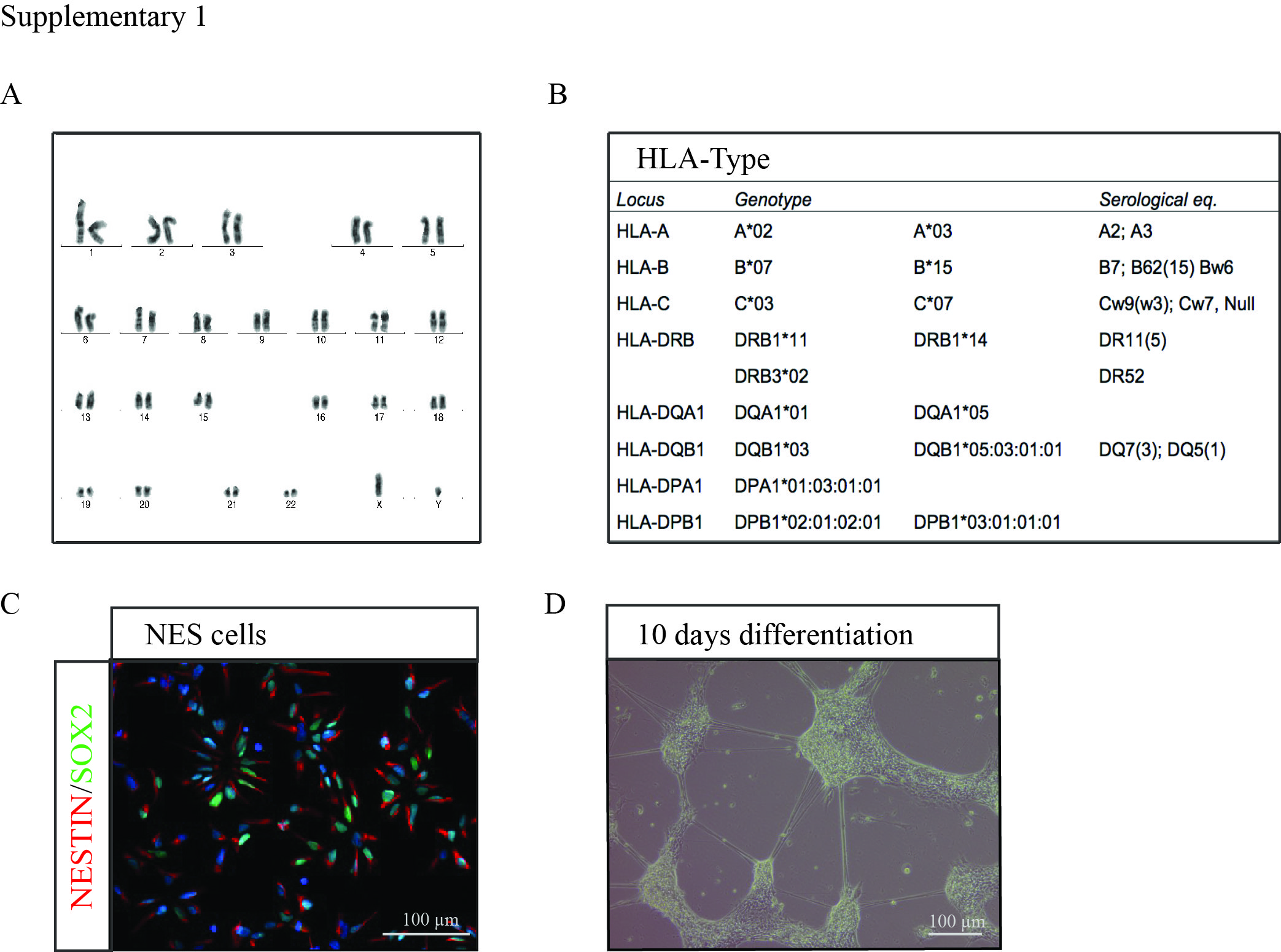

Supplement: Supplementary file 2 [file Image1.JPEG]

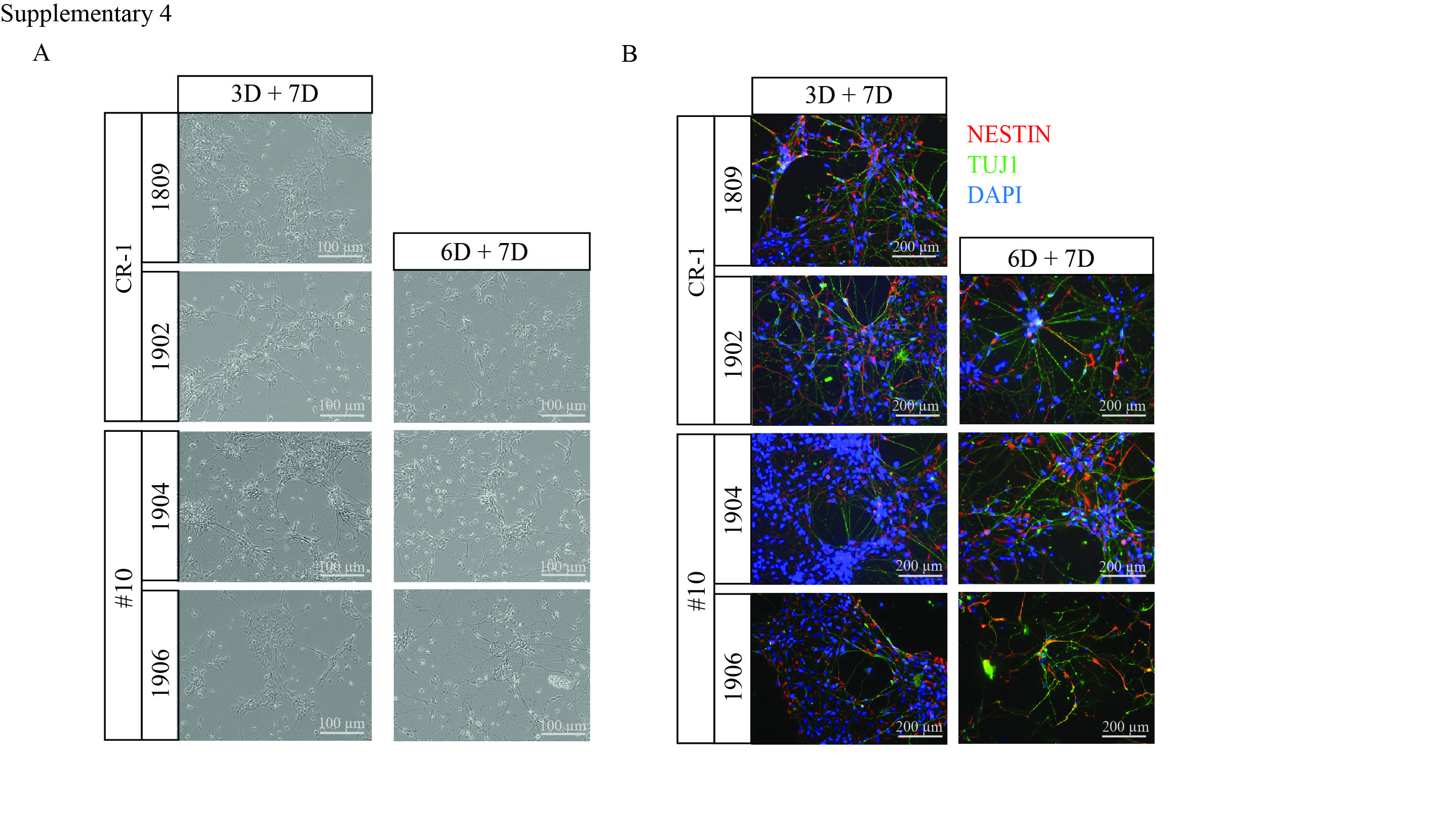

Supplement: Supplementary file 3 [file Image4.JPEG]

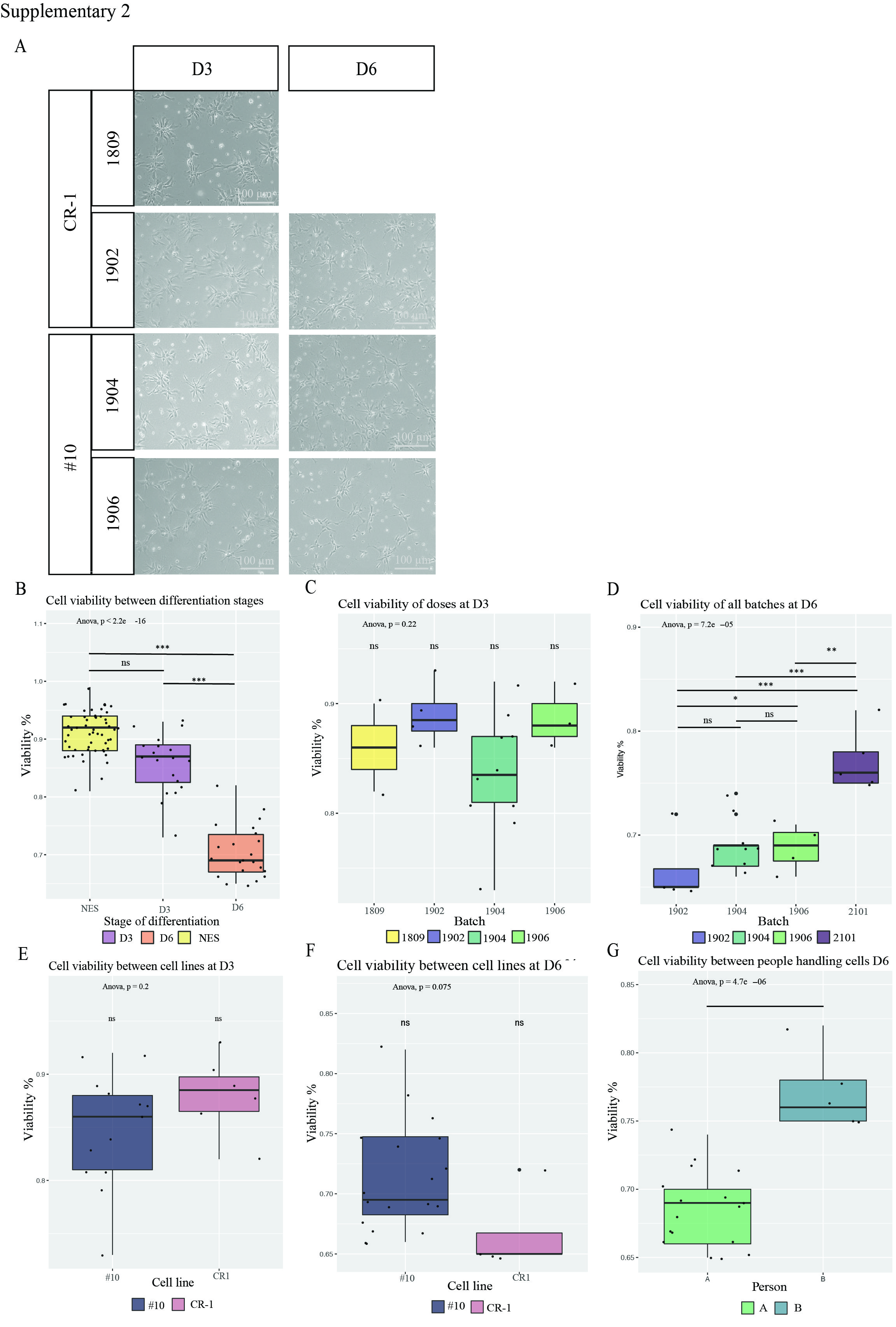

Supplement: Supplementary file 4 [file Image2.JPEG]
